# Mangiferin Attenuates Myocardial Ischemia-Reperfusion Injury via MAPK/Nrf-2/HO-1/NF-*κ*B *In Vitro* and *In Vivo*

**DOI:** 10.1155/2019/7285434

**Published:** 2019-05-13

**Authors:** Kun Liu, Fei Wang, Shuo Wang, Wei-Nan Li, Qing Ye

**Affiliations:** ^1^Department of Cardiothoracic Surgery, Affiliated Hospital of Nantong University, Nantong, China; ^2^Department of Obstetrics and Gynecology, Affiliated Hospital of Nantong University, Nantong, China

## Abstract

The aim of this study was to investigate the cardioprotective effect of mangiferin (MAF) *in vitro* and *in vivo*. Oxidative stress and inflammatory injury were detected in coronary artery ligation in rats and also in hypoxia-reoxygenation- (H/R-) induced H9c2 cells. MAF inhibited myocardial oxidative stress and proinflammatory cytokines in rats with coronary artery occlusion. The ST segment of MAF treatment groups also resumed. Triphenyltetrazolium chloride (TTC) staining and pathological analysis showed that MAF could significantly reduce myocardial injury. In vitro data showed that MAF could improve hypoxia/reoxygenation- (H/R-) induced H9c2 cell activity. In addition, MAF could significantly reduce oxidative stress and inflammatory pathway protein expression in H/R-induced H9c2 cells. This study has clarified the protective effects of MAF on myocardial injury and also confirmed that oxidative stress and inflammation were involved in the myocardial ischemia-reperfusion injury (I/R) model.

## 1. Introduction

Myocardial ischemia-reperfusion injury is the most common cause of death, with more than 6 million deaths worldwide every year [1]. The treatment strategy is aimed at restoring blood flow using thrombolysis or direct angioplasty through a percutaneous coronary intervention (PCI) arterial stent. Paradoxically, in addition to direct ischemic injury, the recovery of blood flow can also cause damage to tissues and further limits the beneficial effects of myocardial reperfusion. This phenomenon, known as reperfusion injury, is associated with the death of cardiac cells, which can survive before reperfusion [2]. The pathogenesis of reperfusion injury involves the interaction of various mechanisms, including the release of vasoconstrictor agents, nonreperfusion, profound inflammatory reactions, apoptosis, and necrosis [3]. During reperfusion, a variety of cytokines, such as interleukin-6 (IL-6) and tumor necrosis factor *α* (TNF-*α*), are released, which caused excessive regional inflammatory reactions and further myocardial damage. Many studies have shown that inhibiting excessive inflammation can reduce the scale of infarction and improve heart dysfunction caused by I/R injury [4].

What is related to ischemia-reperfusion injury is that redox imbalance leads to the activation of activated oxygen and activated nitrogen-mediated signaling pathways. Oxidative stress was considered to be the cause and treatment target of early myocardial ischemia-reperfusion injury [5, 6].

Myocardial infarction leads to ischemic necrosis of the heart tissue, which is the main cause of death. After infarction, proinflammatory immune cells, especially polymorphonuclear leukocytes/monocytes, are rapidly recruited into the heart. Infiltrating neutrophils and macrophages are activated to induce inflammation, oxidative stress, and uncontrolled deposition of extracellular matrix components [7].

To our knowledge, activating NF-*κ*B and MAPK can lead to myocardial ischemia. NF-*κ*B and MAPK pathways have been shown to participate in many physiological functions, including hypertension, heart failure, and myocardial hypertrophy. Various studies have shown that the inhibition of MAPK signal activated nuclear factor erythroid 2-related factor 2 (Nrf-2), which controls the expression of various antioxidant enzymes and second-stage detoxification enzymes, such as heme oxygenase 1 (HO-1). HO-1 is a subtype of heme oxygenase (HO) that plays a central role in cell antioxidant defense. Many reports show that HO-1 plays an important role in promoting myocardial cell survival and protecting myocardial cells from I/R injury [8]. Mangiferin is a xanthone C-glucoside that is a component of many plants in the Cyclopia subfamily such as *Coffea arabica* and *Anemarrhena rhizome*. Studies have found that isomangiferin exhibits a variety of pharmacological properties, such as anti-inflammation and neuroprotection [9]. However, there are few researches to explore whether mangiferin is beneficial for ischemia/reperfusion injury. Therefore, the aim of the present study was to evaluate the effects of mangiferin on ischemia/reperfusion injury and explore its possible mechanism.

## 2. Materials and Methods

### 2.1. Reagents

Mangiferin was purchased from Shanghai Ye Yuan Biotechnology Co. Ltd. (Shanghai, China). Interleukin- (IL-) 6, IL-1*β*, and tumor necrosis factor- (TNF-) *α* ELISA kits were purchased from Elabscience. Myocardial enzymes (CK) and lactate dehydrogenase (LDH) were purchased from Jiancheng Bioengineering Institute (Nanjing, China). All antibodies are provided by Cell Signaling Technology (Danvers, USA).

### 2.2. Animals

Healthy adult male SD rats (about 250 g) are provided by the Animal Center of Nantong University. The living environment of all SD rats is the same. All SD rats are placed at temperatures of 23-25°C and humidity of 55-70%. During this period, all SD rats are raised by standard foods. All are allowed to drink freely. Before the experiment, all the animals were required to fast for 12 hours. All animal operations were approved by Nantong University.

### 2.3. Experimental Protocol I/R

The SD rats were as follows (*n* = 10, 10 rats in each group): sham group, I/R group, I/R+diltiazem (Dil, 20 mg/kg) group, and I/R+MAF (500 mg/kg) group. The sham operation group did not have ischemia-reperfusion but was given normal perfusion at the same time. Rats in the sham operation group and ischemia-reperfusion group were given normal saline for 4 weeks. Rats in Dil and MAF treatment groups were given Dil (20 mg/kg, orally) and MAF (50 mg/kg, orally) for 4 weeks, followed by ischemia-reperfusion.

According to our previous report [10], each healthy SD male rat was carefully weighed and prepared to undergo an intraperitoneal injection (0.5 ml/100 mg) of ethyl carbamate, subject to effective anesthesia. After the rats were successfully anesthetized, the rats were fixed on the operating table in supine position and the rats were carefully connected to a standard lead electrocardiogram instrument to monitor whether the operation indexes were abnormal. During the operation, the experimenter recorded ECG and calculated the heart rate through ECG R wave. At the same time, the experimenter excluded rats with abnormal ECG by monitoring ECG. The trachea of the rat was carefully and bluntly separated and exposed with a scalpel, and immediately after careful incision, mechanical ventilation was given with a ventilator connected to the animal (breathing frequency 60 times/minute, tidal volume 8 ml, frequency 5 : 4). After endotracheal intubation in rats, the left common carotid artery was immediately separated, the artery was internally cannulated, and a pressure sensor was connected to the biological information collection system (BL-410, Nihon Kohden, Japan) to collect information. The chest and chest hair of SD rats were carefully removed, and then the skin was carefully disinfected with iodophor. Then, the following steps were done: open the left chest to expose the heart, peel off the pericardium to find the coronary artery, pass through a small vinyl tube 2 mm away from the left atrial appendage, and make the ligature (4/min wire) form a loop to ligate the coronary artery. LAD ligation induced myocardial ischemia for 30 minutes and reperfusion for 120 minutes. The same operation was used for the sham operation, and LAD only had a ferrule and was not ligated. After the myocardial ischemia-reperfusion operation was completed, the experimenter carefully sutured the muscle and skin of the rat layer by layer and injected a small amount of penicillin or wiped with iodophor at the incision to disinfect the wound and prevent wound infection.

### 2.4. Experimental Protocol of Hypoxia-Reoxygenation (H/R)

According to our previous report [10], taking H9c2 cells growing in the logarithmic growth phase to discard the original culture solution and replace it with anoxic solution saturated with 95% N_2_ and 5% CO_2_ mixed gas in advance, placing the culture plate in a sealed container (37°C) with 95% N_2_ and 5% CO_2_ continuous aeration for 6 h to obtain anoxic solution, replacing the anoxic solution with reoxygenation solution saturated with 95% O_2_ and 5% CO_2_ mixed gas in advance, and placing it in a CO_2_ incubator (5% CO_2_, 95% air, and 37°C temperature) for reoxygenation culture to obtain reoxygenation were carried out.

### 2.5. Cell Viability Assay

According to our previous report [10], H9c2 cardiomyocytes were seeded on a 96-well plate 1 × 10^4^/well. After 6 hours of hypoxia treatment, cells were cultured for 24 hours after reoxidation according to the instructions of the CCK8 detection kit. 10 *μ*l of CCK8 solution was added to the culture medium. The optical density at 450 nm wavelength was measured with a microplate reader and incubated for 2 hours. Secondly, before receiving H/R treatment, the cells hatched at different concentrations of MAF (1, 2, 4, 8, and 16 *μ*M) and were then evaluated according to the aforementioned cell viability. In other index tests, H9c2 cells were cultured in 2 × 10^5^ cells/well for 24 hours. After pretreatment with or without MAF (8 *μ*M), it was used for the detection of high-concentration therapy, cell supernatant, and cell collection for other indicator detection.

### 2.6. Electrocardiogram (ECG) Measurement

According to our previous report [10], the following data were observed: SD rats were given intraperitoneal injection anesthesia (50 mg/kg) with pentobarbital sodium according to their body weight; then, the rats were fixed in the back position on the experimental board; the neck and chest were covered with skin, then disinfected with iodophor; sterile hole towels were laid; needle-shaped ECG electrodes were inserted under the skin of the rats' limbs, connected with the electrocardiograph; and ECG changes were monitored and recorded.

### 2.7. Determination of Myocardial Infarct Size

According to our previous report [10], the following steps should be performed: quickly take out the rat heart, wash away blood stains in saline water placed on ice, remove irrelevant tissues above the ligature with a blade, then freeze the heart in a -20°C refrigerator for 30 min, place it in a 1% TTC solution, and stand it in 37°C water for 15 min in the dark. Myocardial infarction area is the ratio of myocardial infarction to the risk area by a percentage of risk the area (infarction area/risk area).

### 2.8. Determination of Inflammatory Cytokines in Serum, in the LV Myocardium, and in Cell Supernatant

According to the instructions of the manufacturer, the contents of IL-6, IL-1*β*, and TNF-*α* in serum, in the LV myocardium, and in cell supernatant were determined using the ELISA kits.

### 2.9. Detection of Myocardial Enzymes

Myocardial enzymes are indicators of myocardial necrosis. Blood was collected from the left common carotid artery and centrifuged at 3500 rpm for 10 minutes; supernatant was collected and measured with the CK and LDH kits.

### 2.10. SOD and MDA Assay in Serum and Cell Supernatant

Blood was collected from the left common carotid artery and centrifuged at 3500 rpm for 10 minutes; supernatant was collected and measured with the SOD and MDA kits. After H/R, cell supernatants were collected and SOD and MDA in cell supernatants were detected by the SOD and MDA detection kits.

### 2.11. Histological Examination of the Myocardium

According to our previous report [10], the following steps should be followed: carefully remove the rat's heart and place it in precooled PBS, carefully remove connective tissue and adipose tissue, and quickly freeze the heart tissue in isopentane. This process should prevent ice crystals from forming, and then we carefully cut the tissue into slices of about 4 Mn with a freezing microtome, followed by putting the cut tissue in the fixing solution for about 10-30 s and washing it with water after fixing for 1-2 s. After that, it was dyed with hematoxylin for 0. 5 min and washed with running water. Then, 1% hydrochloric acid alcohol was used to differentiate for 1-3 s, weak ammonia water was used to soak for 5-10 s, 0.5% eosin solution was used to dye for 30-60 s, and ethanol with different concentrations was used to wash for 2 s in turn. The final gum (formulated as neutral) seal was observed under an optical microscope.

### 2.12. Western Blot

According to our previous report [10]: the total protein of the heart tissue and H9c2 cells of each group was extracted from RIPA lysol and 12,000 rpm centrifugation of 15 min was collected. The BCA kit was used to quantify each protein. Each group of protein samples was electrophoresis with SDS-polyacrylamide gel and then transferred to the PVDF membrane. The PVDF membrane is then placed in 5% skim milk and closed 2 h. After closing 2 h, the PVDF membrane is placed in the corresponding antibodies (p-JNK, JNK, p-ERK, ERK, p-P38, P38, HO-1, Nrf-2, p-P65, P65, caspase-3, caspase-9, Bax, and Bcl-2) and incubated overnight at 4°C. We washed the membrane with TBST 4 times the next day, 8 minutes each time. The PVDF membrane is then placed in a direactance solution and incubated at room temperature for 2 h in the shaker. Then, we removed the PVDF membrane and washed it with TBST 4 times, 8 minutes each time. Gel imaging system is used to expose and scan strips to analyze the gray value of each band.

### 2.13. Statistical Analysis

Data are expressed as means ± SDs of at least three separate experiments. Statistical comparisons between experimental groups were performed by ANOVA with the Tukey multiple comparison test. A value of *p* < 0.05 was considered statistically significant.

## 3. Results

### 3.1. Effect of MAF on Myocardial Infarct Size

**Figure 1:**
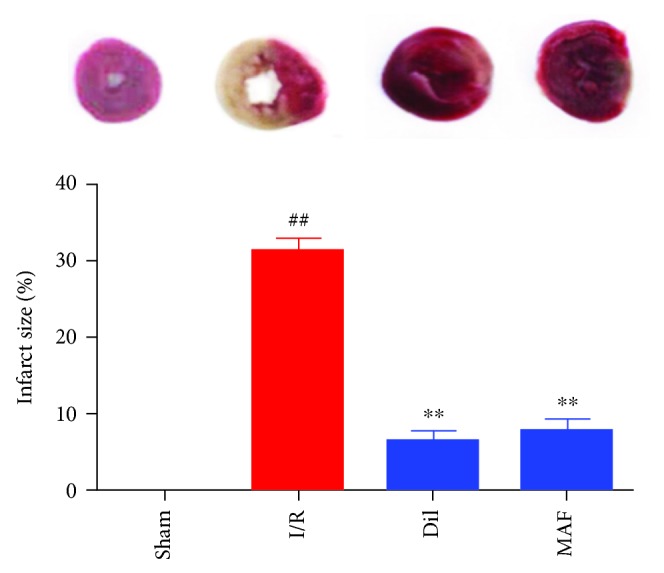
Effects of MAF on myocardial infarct size. The data are expressed as mean values ± SDs. ^#^*p* < 0.05 and ^##^*p* < 0.01 compared with the control group. ^∗^*p* < 0.05 and ^∗∗^*p* < 0.01 compared with the I/R group.

As revealed in Figure 1, there was scarce infarct damage in the control group of rats, whereas the percent of infarct size in the I/R group was obviously higher than that in the control group. On the contrary, treatment with Dil (10 mg/kg) and MAF (50 mg/kg) notably reduced the infarct size.

### 3.2. Effect of MAF on Myocardial Histology

**Figure 2:**
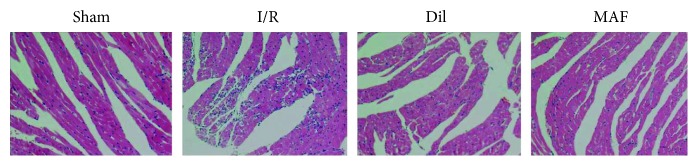
Effect of MAF on myocardial histology. Original magnification (×200).

As illustrated in Figure 2, the sections of myocardial tissues in the sham group presented obvious integrity of the myocardial membrane, a normal myofibrillar structure with striations, branched appearance, and continuity with adjacent myofibrils under light microscopy. Hearts subjected to heart ligation operation exhibited large numbers of infiltrating inflammatory cells, myocardial cell swelling, degeneration, cardiac necrosis, and loss of transverse striations. MAF (50 mg/kg) and Dil (10 mg/kg) significantly improved the described pathological changes above.

### 3.3. Effect of MAF on Cell Viability in H9c2 Cells

**Figure 3:**
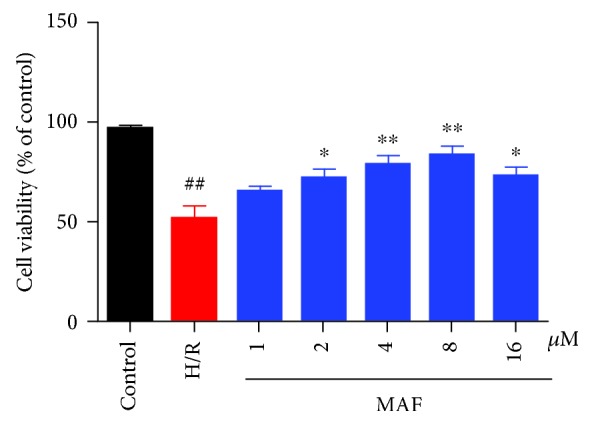
Effect of MAF on cell viability in H9c2 cells exposed to hypoxia for 6 h, followed by reoxygenation (by CCK8 assay). The data are expressed as mean values ± SDs. ^#^*p* < 0.05 and ^##^*p* < 0.01 compared with the control group. ^∗^*p* < 0.05 and ^∗∗^*p* < 0.01 compared with the H/R group.

As illustrated in Figure 3, cells were exposed to hypoxia for 6 h, followed by reoxygenation for 24 h. Then, cell viability was detected at 24 h after reoxygenation by CCK8 assay. 6 h of hypoxia caused a decrease of cell viability, while MAF increased the cell viability of H9c2 cells.

### 3.4. Effects of MAF on ST Segment Elevation

**Figure 4:**
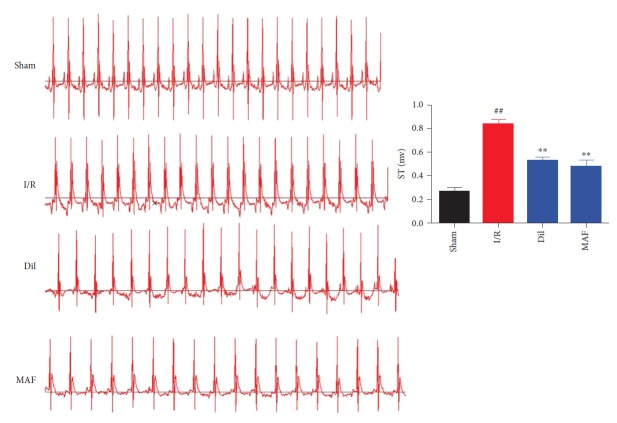
Effects of MAF on ST segment elevation. The data are expressed as mean values ± SDs. ^#^*p* < 0.05 and ^##^*p* < 0.01 compared with the control group. ^∗^*p* < 0.05 and ^∗∗^*p* < 0.01 compared with the I/R group.

As illustrated in Figure 4, the ST segment was dramatically elevated and R amplitude was decreased in the I/R group compared with those in the control group. The results represented that the I/R damage model was well established. MAF and Dil obviously ameliorated the above-described conditions, which partially evidenced the protective effects.

### 3.5. Effects of MAF on Inflammatory Cytokines in Serum, in the LV Myocardium, and in Cell Supernatant

**Figure 5:**
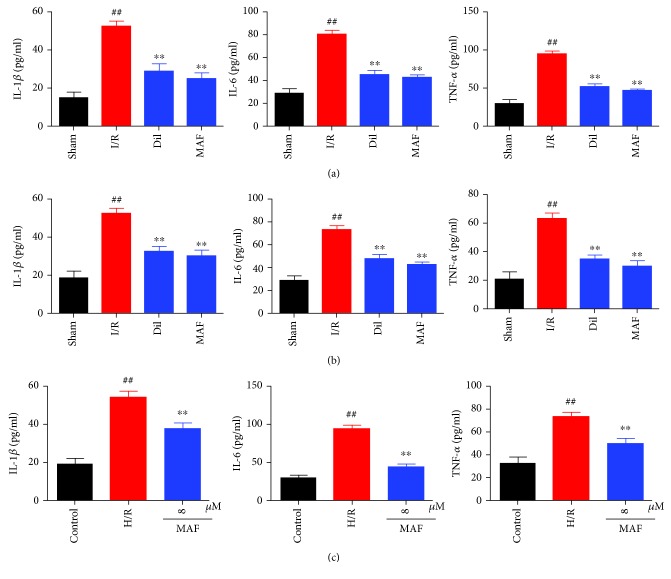
Effects of MAF on inflammatory cytokines in serum (a), in the LV myocardium (b), and in cell supernatant (c). The data are expressed as mean values ± SDs. ^#^*p* < 0.05 and ^##^*p* < 0.01 compared with the control group. ^∗^*p* < 0.05 and ^∗∗^*p* < 0.01 compared with the I/R group and H/R group.

As illustrated in Figure 5, the serum, LV myocardium, and cell supernatant levels of TNF-*α*, IL-6, and IL-1*β* were assessed. It was proven that there were significant increases of the serum, LV myocardium, and cell supernatant levels of TNF-*α*, IL-6, and IL-1*β* in the I/R group. MAF or Dil decreased the serum, LV myocardium, and cell supernatant levels of TNF-*α*, IL-6, and IL-1*β*.

### 3.6. Effects of MAF on Cardiac Marker Enzymes

**Figure 6:**
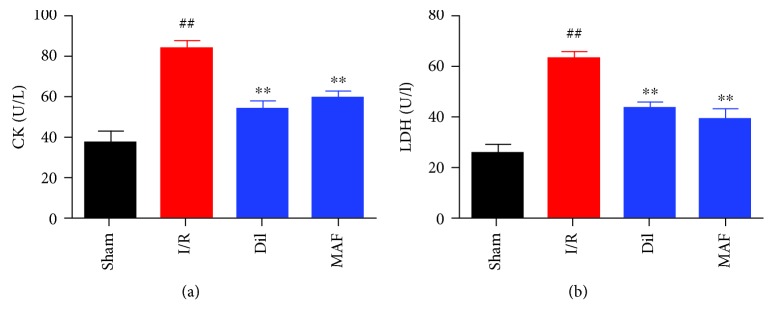
Effects of MAF on cardiac marker enzymes. The data are expressed as mean values ± SDs. ^#^*p* < 0.05 and ^##^*p* < 0.01 compared with the control group. ^∗^*p* < 0.05 and ^∗∗^*p* < 0.01 compared with the I/R group.

As depicted in Figure 6, the levels of CK and LDH were obviously increased in I/R animals compared with those in sham rats, while MAF and Dil significantly decreased CK and LDH compared with the I/R group.

### 3.7. Effects of MAF on SOD and MDA in Serum and Cell Supernatant

**Figure 7:**
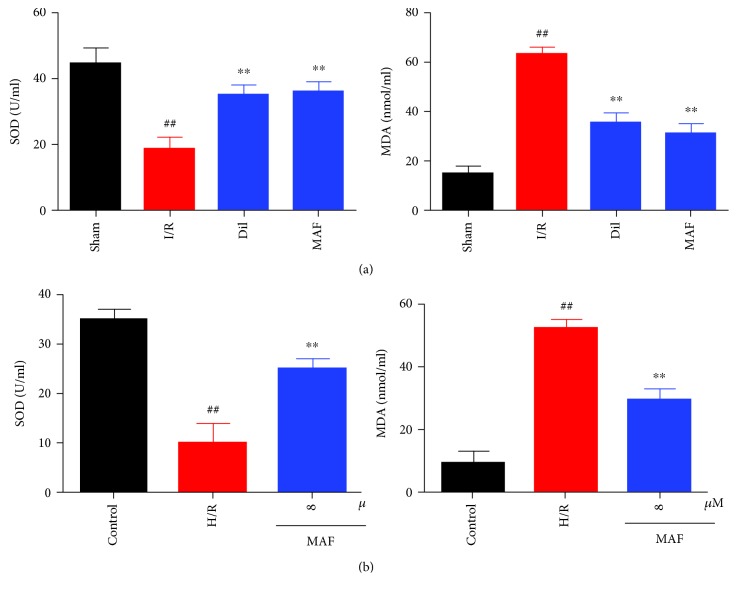
Effects of MAF on SOD and MDA in serum (a) and cell supernatant (b). The data are expressed as mean values ± SDs. ^#^*p* < 0.05 and ^##^*p* < 0.01 compared with the control group. ^∗^*p* < 0.05 and ^∗∗^*p* < 0.01 compared with the I/R group and H/R group.

As shown in Figure 7, exposure to I/R or H/R displayed a strikingly elevated MDA level and decreased SOD. By contrast, the treatment with MAF or Dil evidently decreased the MDA content and increased the SOD level.

### 3.8. Effects of MAF on the MAPK/Nrf-2/HO-1/NF-*κ*B Pathway in I/R-Induced Rats

**Figure 8:**
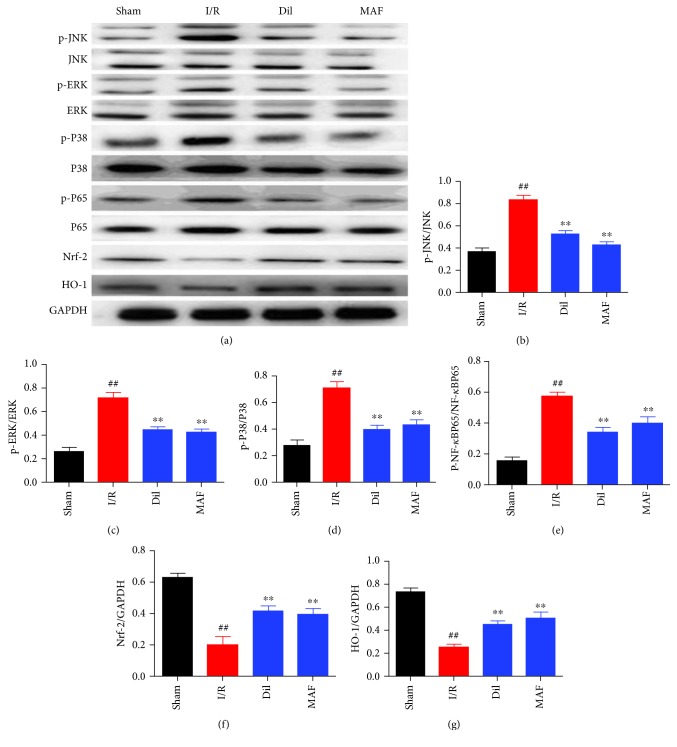
Effects of MAF on the MAPK/Nrf-2/HO-1/NF-*κ*B pathway in I/R-induced rats. The data are expressed as mean values ± SDs. ^#^*p* < 0.05 and ^##^*p* < 0.01 compared with the control group. ^∗^*p* < 0.05 and ^∗∗^*p* < 0.01 compared with the I/R group.

We found that the levels of p-JNK (the ratio is 63.8% compared with JNK), p-ERK (the ratio is 43.3% compared with ERK), p-P38 (the ratio is 41.9% compared with P38), and p-P65 (the ratio is 54.7% compared with P65) were significantly upregulated and the levels of Nrf-2 and HO-1 were significantly downregulated in heart tissues after I/R stimulation. However, MAF obviously restored these situations, and diltiazem has similar effect on the APK/Nrf-2/HO-1/NF-*κ*B pathway in I/R-induced rats (Figure 8).

### 3.9. Effects of MAF on the MAPK/Nrf-2/HO-1/NF-*κ*B Pathway in H/R-Induced H9c2 Cells

**Figure 9:**
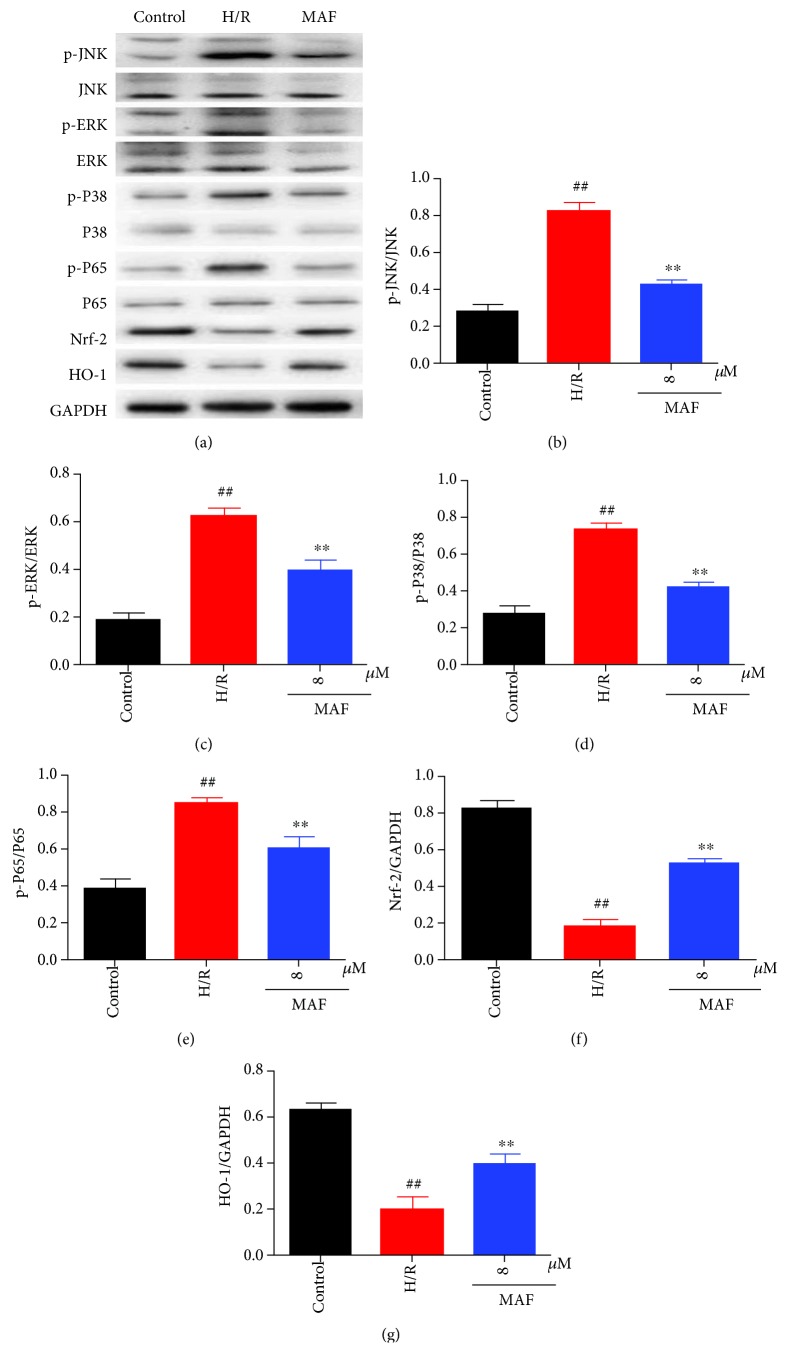
Effects of MAF on the MAPK/Nrf-2/HO-1/NF-*κ*B pathway in H/R-induced H9c2 cells. The data are expressed as mean values ± SDs. ^#^*p* < 0.05 and ^##^*p* < 0.01 compared with the control group. ^∗^*p* < 0.05 and ^∗∗^*p* < 0.01 compared with the H/R group.

We found that the levels of p-JNK (the ratio is 56.3% compared with JNK), p-ERK (the ratio is 44.2% compared with ERK), p-P38 (the ratio is 58.7% compared with P38), and p-P65 (the ratio is 46.5% compared with P65) were significantly upregulated and levels of Nrf-2 and HO-1 were significantly downregulated in H9c2 cells after H/R stimulation. However, MAF obviously restored these situations, and diltiazem has similar effect on the MAPK/Nrf-2/HO-1/NF-*κ*B pathway in H/R-induced H9c2 cells (Figure 9).

### 3.10. Effects of MAF on Apoptosis-Related Protein in I/R-Induced Rats

**Figure 10:**
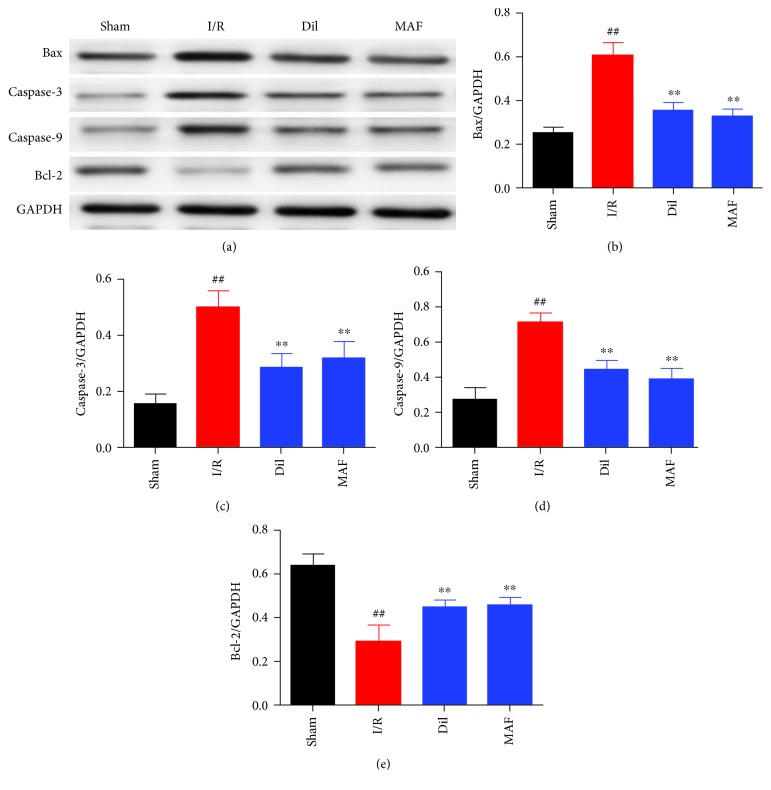
Effects of MAF on apoptosis-related proteins in the myocardium I/R-induced rats. The data are expressed as mean values ± SDs. ^#^*p* < 0.05, ^##^*p* < 0.01 compared with the sham group. ^∗^*p* < 0.05 and ^∗∗^*p* < 0.01 compared with the I/R group.

As shown in Figure 10, exposure to I/R displayed strikingly elevated levels of Bax, caspase-3, and caspase-9 and a decreased level of Bcl-2. By contrast, the treatment with MAF and Dil evidently decreased the levels of Bax, caspase-3, and caspase-9 and increased the Bcl-2 level (Figure 10).

## 4. Discussion

At present, there are many interventions for cardiovascular diseases, such as thrombolysis and coronary artery bypass surgery for coronary heart disease. Although the mortality rate has dropped significantly, the ischemic reperfusion injury damage associated with these treatments makes the results less satisfactory. Therefore, in medical research, prevention and treatment of ischemic reperfusion heart tissue are needed. In the current research, MAF therapy has improved ST, reduced the myocardial infarction area, reduced CK-MB and LDH, improved myocardial pathological changes, and restored inflammation and oxidative stress-related pathways in the myocardial ischemia/reperfusion environment.

At present, there are several kinds of drugs for treating myocardial ischemia: the first category is calcium channel blockers such as nifedipine and diltiazem. The second category is antioxidant free radical generators such as vitamin C. The third category is energy mixture creatine phosphate. The fourth category is anti-white blood cell aggregation such as ulinastatin. Among them, the first type of the calcium ion blocker is the most commonly used drug for treating myocardial ischemia and is also the most widely used drug for treating myocardial ischemia. Other researchers have also reported the therapeutic effect of diltiazem on myocardial ischemia-reperfusion injury [2, 11, 12], which is the basis for our selection of diltiazem as a positive drug in this experiment. On the other hand, Agustini et al. [13] found that mangiferin can protect azithromycin-induced myocardial injury by adjusting the calcium ion level, which indicates that mangiferin has regulatory effect on calcium ion-mediated injury. Based on the above two aspects, the calcium ion blocker diltiazem was selected as the positive drug in this study when studying the mechanism of mangiferin on myocardial ischemia-reperfusion injury.

According to reports, the MAPK pathway is necessary for cell survival and apoptosis [14–16]. In this regard, we assume that MAF's protective effect on the H9c2 cardiomyocytes is related to the mechanism of the MAPK signal pathway. As expected, our results shown that MAF therapy selectively increased the inhibition of ERK 1/2, JNK, and p38 in rats and H9c2 cells. As the downstream molecules of MAPK, NF-*κ*B plays an essential role in the regulation of inflammatory and apoptotic progression in myocardial ischemia [17, 18]. The activation of NF-*κ*B is triggered by the phosphorylation and degradation of the I*κ*B*α* [19]. NF-*κ*B governs the mediation of inflammatory cytokine production, oxidative stress, and apoptotic reaction [20]. There is evidence that the MAPK/NF-*κ*B pathway is involved in myocardial ischemia-reperfusion injury in vivo and in vitro [21]. Our results confirmed that in the I/R model, the levels of p-JNK, p-ERK, p-P38, and p-P65 were increased compared with those in the sham group. MAF and Dil could decrease the levels of p-JNK, p-ERK, p-P38, and p-P65 compared with the I/R group. In H9c2 cells after H/R stimulation, the levels of p-JNK, p-ERK, p-P38, and p-P65 were increased compared with those in the control group. MAF could decrease the levels of p-JNK, p-ERK, p-P38, and p-P65 compared with the H/R group.

Myocardial ischemia/reperfusion can cause cytokines such as TNF-*α*, IL-6, and IL-1*β*. Infarct increases cell permeability, enhances caspase cascade reaction, and leads to apoptosis of a large number of myocardial cells. TNF-*α* is mainly secreted by macrophages, which may promote inflammatory cascade reaction by increasing the release of other proinflammatory cytokines and affecting neutrophil recruitment. As an important cytokine in inflammation, IL-6 plays an important role in inflammation induced by I/R. IL-1*β* can induce the release of other inflammatory mediators, increase the expression of cell adhesion factors, and promote the adhesion of neutrophils to endothelial cells. The experimental results showed that MAF and Dil successfully reduced the levels of tumor necrosis factor-*α*, interleukin-6, and interleukin-1*β* in rats. In H9c2 cells after H/R stimulation, the levels of TNF-*α*, IL-6, and IL-1*β* were increased compared with those in the sham group. MAF could decrease the levels of TNF-*α*, IL-6, and IL-1*β* compared with the H/R group.

Several studies have shown that the inhibition of MAPK activation can activate Nrf-2 and then the regulation of HO-1 [22]. Activated Nfr-2 is released from Keap1, an inhibitor of Nrf-2, and transferred to the nucleus, where it binds to the antioxidant reaction element (ARES) and accelerates the expression of HO1. HO-1 is one of the second stage antiseptics and is reported to play a key role in the prevention of injury caused by I/R [23, 24]. Our results confirmed that in the I/R model, the levels of Nrf-2, HO-1, and SOD were decreased and the level of MDA was increased compared with that in the sham group. MAF and Dil could increase the levels of Nrf-2, HO-1, and SOD and decrease the level of MDA compared with the I/R group. In H9c2 cells after H/R stimulation, the levels of p-JNK, p-ERK, p-P38, and p-P65 were increased compared with those in the control group. MAF could increase the levels of Nrf-2, HO-1, and SOD and decrease the level of MDA compared with the H/R group.

**Figure 11:**
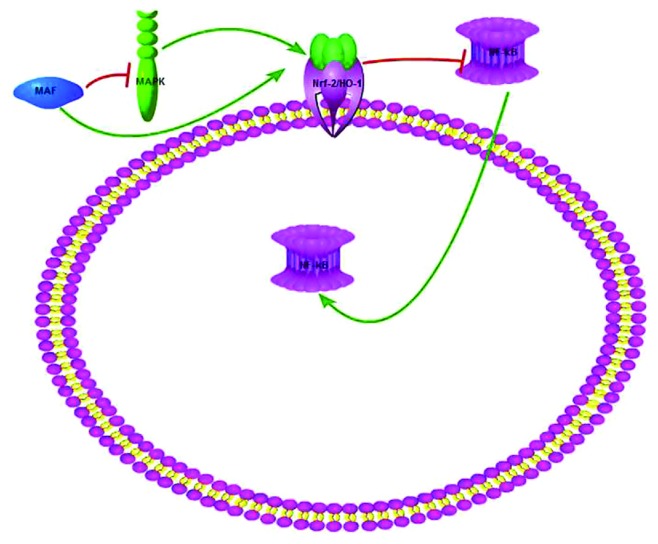
The schematic outline depicts the proposed mechanism of MAF on I/R via MAPK/Nrf-2/HO-1/NF-*κ*B pathway.

These findings suggested that MAF could be used as a new protective agent by inhibiting oxidative stress and inflammation in vitro and in vivo (the mechanism is shown in Figure 11). Further researches are warranted for more details in the future.

## Data Availability

The data used to support the findings of this study are available from the corresponding author upon request.
